# svdPPCS: an effective singular value decomposition-based method for conserved and divergent co-expression gene module identification

**DOI:** 10.1186/1471-2105-11-338

**Published:** 2010-06-22

**Authors:** Wensheng Zhang, Andrea Edwards, Wei Fan, Dongxiao Zhu, Kun Zhang

**Affiliations:** 1 Department of Computer Science, Xavier University of Louisiana, 1 Drexel Drive, New Orleans LA 70125, USA; 2 IBM T.J.Watson Research, 19 Skyline Drive, Hawthorne NY 10532, USA; 3 Department of Computer Science, University of New Orleans, 2000 Lakeshore Drive, New Orleans LA 70122, USA

## Abstract

**Background:**

Comparative analysis of gene expression profiling of multiple biological categories, such as different species of organisms or different kinds of tissue, promises to enhance the fundamental understanding of the universality as well as the specialization of mechanisms and related biological themes. Grouping genes with a similar expression pattern or exhibiting co-expression together is a starting point in understanding and analyzing gene expression data. In recent literature, gene module level analysis is advocated in order to understand biological network design and system behaviors in disease and life processes; however, practical difficulties often lie in the implementation of existing methods.

**Results:**

Using the singular value decomposition (SVD) technique, we developed a new computational tool, named svdPPCS (**SVD**-based **P**attern **P**airing and **C**hart **S**plitting), to identify conserved and divergent co-expression modules of two sets of microarray experiments. In the proposed methods, gene modules are identified by splitting the two-way chart coordinated with a pair of left singular vectors factorized from the gene expression matrices of the two biological categories. Importantly, the cutoffs are determined by a data-driven algorithm using the well-defined statistic, SVD-p. The implementation was illustrated on two time series microarray data sets generated from the samples of accessory gland (ACG) and malpighian tubule (MT) tissues of the line W^118 ^of *M. drosophila*. Two conserved modules and six divergent modules, each of which has a unique characteristic profile across tissue kinds and aging processes, were identified. The number of genes contained in these models ranged from five to a few hundred. Three to over a hundred GO terms were over-represented in individual modules with FDR < 0.1. One divergent module suggested the tissue-specific relationship between the expressions of mitochondrion-related genes and the aging process. This finding, together with others, may be of biological significance. The validity of the proposed SVD-based method was further verified by a simulation study, as well as the comparisons with regression analysis and cubic spline regression analysis plus PAM based clustering.

**Conclusions:**

svdPPCS is a novel computational tool for the comparative analysis of transcriptional profiling. It especially fits the comparison of time series data of related organisms or different tissues of the same organism under equivalent or similar experimental conditions. The general scheme can be directly extended to the comparisons of multiple data sets. It also can be applied to the integration of data sets from different platforms and of different sources.

## Background

Comparative analysis of gene expression profiling of multiple biological categories, such as different species of organisms or different kinds of tissue, promises to enhance the fundamental understanding of the universality as well as the specialization of molecular biochemistry mechanisms and related biological themes. Recent studies in this field have led to remarkable results in providing insights to the transcriptional programs of several organisms [1-5]. Grouping genes with a similar expression pattern or exhibiting co-expression together is a starting point in the analysis of gene expression data. In recent literature, gene module level analysis is advocated in order to understand biological network design and system behaviors in diseases and life processes. Various statistical methods, such as clustering algorithms [5], matrix decomposition techniques [6,7], topology network [8,9], and the procedure of gene specific model analysis followed by gene clustering (or grouping) [10] have been proposed to identify the individually defined gene modules without much consistency.

Singular value decomposition (SVD) is a well-known matrix factorization method that has been widely applied in the analysis of microarray data [11-17]. Assume that the data matrix is in the form with genes in rows and arrays in columns, the profiles of the leading right singular vectors of the decomposition (eigengenes) suggest the fundamental gene expression patterns across the arrays, which in turn represent a biological theme if the data is well organized [18]. The squares of the non-negative singular values represent the relative importance of the corresponding patterns. The left singular vectors contain elements which can be used as confidence scores for separating genes with the same or similar pattern from others [6,17]. Given a cutoff and a direction (positive or negative), we can extract a gene group which is naturally a co-expression cluster. Such a cluster can also be viewed as a functional module related to the addressed biological theme, such as the aging process analyzed in this paper.

A main challenge in implementing SVD-based module identification algorithms is how to choose the cutoffs. One naïve method is to test the gradient values (such as 0.1, 0.2, ...) and then the decision is made according to the results of functional analysis and comparison. However, practical difficulties often lie in choosing the methods for functional analysis and comparison of gene sets. Another method indirectly decides the cutoffs by specifying the module size or the gene proportion assigned to each module. A variant of the latter method is to set the cutoff at a magnitude equivalent to *k *(such as 3) multiplies the standard deviation of the vector elements [14]. These methods are widely used but cannot guarantee insights in both the statistical and biological aspects of the cluster. The "gene shaving" algorithm [13] represents a substantial advance in addressing this challenge. It was designed to optimize the size of a gene cluster by maximizing the Gap statistic. Gap is defined as the difference of the ratio of the between- and within- "mean" gene variances of the cluster and the corresponding ratio of a cluster defined from a null data set. However, in the calculation of between "mean" gene variance, the effects of random arrays and sample groups (or time points for temporal data) can be ambiguous. Furthermore, the sizes of clusters are not directly considered in Gap statistic calculation.

In this paper, we developed a novel computational tool, named svdPPCS (SVD-based Pattern Pairing and Chart Splitting), to identify the conserved and divergent co-expression modules of two sets of microarray experiments. Our definition of gene modules is proposed in the spirit of Ihmels et al. [6]. The conserved module is defined as a gene group which shows similar co-expression patterns across a set of experimental conditions or a biological theme in the two addressed biological categories. The divergent module is defined as a gene group which demonstrates a co-expression pattern only in one of the two biological categories or whose patterns in them are different. The method is novel and important in that: (1) the conserved and divergent modules are defined in the context of the addressed biological theme, such as in the aging process; (2) the modules are identified through splitting the two-way chart coordinated with a pair of left singular vectors factorized from the gene expression matrices of the two biological categories; and (3) the cutoffs are determined by a data-driven algorithm using a well-defined statistic. The proposed scheme is comparable to the widely-used procedure of gene-specific model analysis followed by gene clustering. The advantages of svdPPCS over this routine practice include: (1) the correlations among genes can be considered in the initial stage; (2) the complicated patterns can be easily captured; and (3) the implementation is less subject to the restrictions on the number of time points of temporal microarray data. The proposed method was showcased with two time series microarray data sets generated from the samples of accessory gland (ACG) and malpighian tubule (MT) of wild-type line W^118 ^of *M. drosophila*. The modules identified by svdPPCS were compared with those identified using two alternate methods. A simulation study was further conducted to validate the efficacy of the SVD-based method in data with more complicated patterns.

## Results

### General scheme of svdPPCS

**Figure 1:**
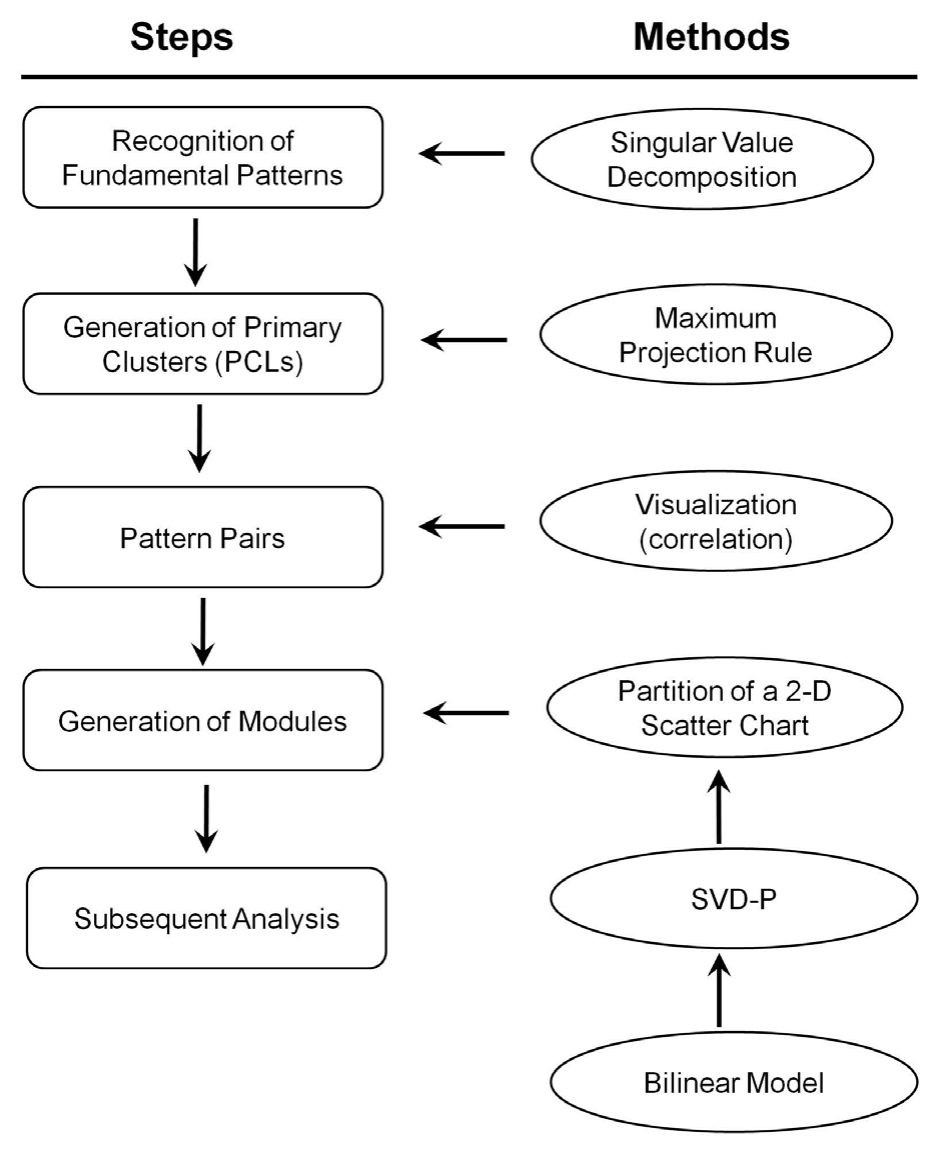
**Flow diagram of svdPPCS**.

The svdPPCS method for identifying conserved and divergent modules across two biological categories is depicted in Figure 1. It includes four steps, namely, recognition of fundamental patterns, generation of primary clusters (PCLs), pattern pairing, and extraction of gene modules. In this section, we present this general scheme in combination with the comparative study of two kinds of tissue of *M. drosophila*. Each of the time series gene expression data sets contains 10 arrays representing 5 time points (see the section of **Study on M. drosophila data **for details). The techniques to calculate SVD-p and to determine the cutoffs are described subsequently.

**Figure 2:**
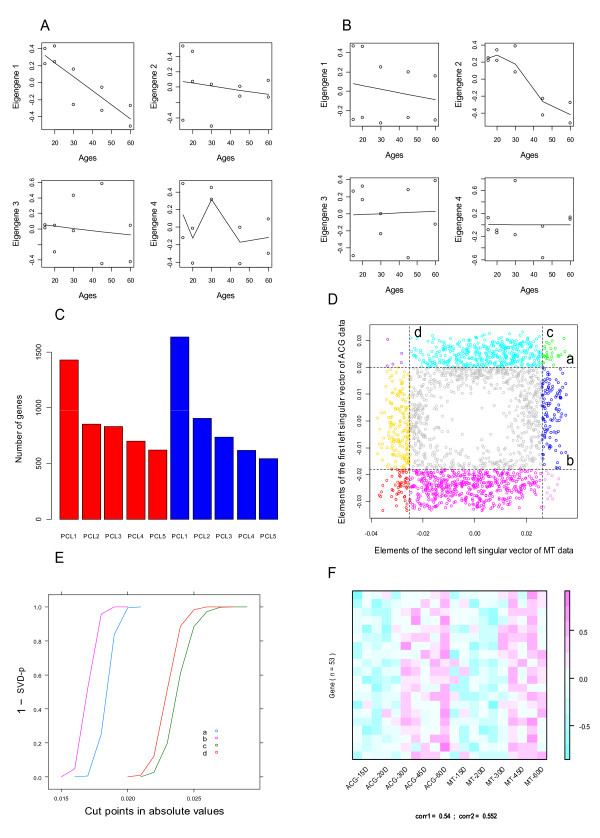
**Illustration of the implementation of svdPPCS**. **A: **The plots of the first four eigengenes of ACG data across ages. **B: **The plots of the first four eigengenes of MT data across ages. **C**: The distribution of genes among the five primary clusters (PCLs) based on the maximum projection rule for ACG data (red) and MT data (blue). **D**: The identification of co-expression modules by splitting a two-way chart. **E: **Determine the cutoffs ***a, b, c***, and ***d ***based on SVD-p. **F**: The heatmap of a conserved gene module.

First of all, the singular value decomposition (SVD) of the *n *× *m *matrix measuring *n *genes and *m *arrays is calculated individually for each data set. The fundamental patterns of biological importance [3,12] hidden in the data are recognized through the visualization of the right singular vectors (eigengenes) of the decompositions. In the *M. drosophila *data, the arrays were previously arranged according to the ages of the flies. Thus, the biological significance of an eigengene regarding the aging process was suggested by the profile of its plot versus arrays (ages). As shown in Figures 2A and 2B, the first eigengene of the ACG data and the second eigengene of the MT data are meaningful because their plots demonstrated apparent structures.

In the second step, primary clusters (PCLs) are generated according to the projections of the gene expression vectors across the arrays onto the eigengenes. That is, if the magnitude of the projection of gene *i *onto eigengene *j *is the largest among all the *m *projections, it will be assigned to the *j^th ^*PCL. For each data set, the genes are divided into *k *(<=*m*) mutually exclusive PCLs. Each PCL is characterized by the profile of the corresponding eigengene. The number of genes assigned to these PCLs is approximately proportional to the magnitudes of the corresponding eigenvalues. The PCL corresponding to the leading fundamental pattern contains the largest proportion of genes. In the analysis of the two *M. drosophila *data sets, we emptied the PCLs corresponding to the eigengenes 6-10, and allocated their genes into the remaining PCLs. Figure 2C shows the distribution of the numbers of genes in the five non-empty PCLs on ACG and MT data, respectively. This step is optional and can be skipped in the scenario that only the leading eigengene holds the pattern of biological importance in both data sets.

In the third step, the similarity of the fundamental patterns between the two data sets is assessed through the visualization or correlation analysis of their right singular vectors. Based on the assessment, one or multiple pattern pairs are established. In the *M drosophila *data, the first eigengene of the ACG subset and the second eigengene of the MT subset had a high correlation (r = 0.88, p < 0.01), thus they formed a pattern pair which can provide biological insights to both tissue kinds. Corresponding to the pattern pair is a couple of primary clusters consisting of the genes to which the contribution of the two eigengenes was the largest among all eigengenes. This process can be directly extended to the comparisons of multiple data sets.

In the fourth step, the genes in either of these two PCLs are mapped onto a coordination system with the two left singular value vectors corresponding to the pattern pair as the x-axis and y-axis, respectively. Given two sets of cutoffs, (***a***, ***b***) and (***c***, ***d***), the genes can be divided into at most nine sub-clusters as shown in Figure 2D. The genes contained in the sub-cluster at the center of Figure 2D will be excluded from further study, and the remaining non-empty sub-clusters are called modules. In the example analysis, two conserved modules (green and red) and six divergent modules (cyan, blue, golden, magenta, purple, and violet) were identified. The cutoffs are determined by a data-driven algorithm using the well-defined statistic, SVD-p. Figure 2E shows that SVD-p decreases gradually toward zero with the increase of the magnitudes (absolute values) of the cutoffs. The employed algorithm is presented in the section of **Determination of cutoffs**.

An additional step is the visualization of the results. As shown in Figure 2F, for each pattern pair we can have a heatmap of 1-8 plots. Each plot is characterized with the unique co-expression pattern of a gene module across the arrays and tissue kinds.

It should be noted that a special data polishing procedure is usually required prior to SVD analysis. In the *M. drosophila *data, we firstly centralized the gene expression by columns and scaled the new values with the standard deviations. After that, row-wide centralization and scaling (with the square roots of inner products rather than the standard deviation) were conducted. The process was repeated twice. Such a two-way centralization has been shown to be effective in unraveling the patterns hidden in a data matrix [12,19].

#### A note on the second step

Reintroducing the genes of the discarded primary clusters (corresponding to the less significant eigengenes) is tricky at this step. The initial purpose is to reduce the potential loss of information due to the exclusion of these genes. However, at the same time, this procedure can introduce undesirable noise to the receiving clusters. Therefore, it should be cautious of doing so. More specifically, the reintroduction needs to be conducted based on the projections of the same gene onto different eigengenes. For example, suppose gene *X *has the largest projection onto eigengene-10 and the second largest projection onto the eigengene-2, *X *would be reintroduced into the PCL-2 when PCL-10 is discarded. In general, we recommend (1) keeping the primary clusters corresponding to the first *k *eigengenes with the total contribution up to 60%; and (2) only the eigengene(s) with the pattern(s) related to the addressed biological theme, such as the aging process, are considered in deriving the defined co-expression modules.

#### A note on the third step

The generation of pattern pairs included two steps. (1) From each data set, identify the eigengenes with the patterns being meaningful for the addressed biological themes, such as the aging process; and (2) Select all pattern pairs by calculating the correlations between the eigenvectors of the two data sets and conducting significance tests. If the experimental conditions of the two data sets are simply similar to each other but not fully equivalent, the generation of the pattern pairs should be based on the visualization of the eigengenes.

### Calculation of SVD-p

SVD-p is defined to measure the importance of the leading latent factor (representing the leading pattern) in explaining the variance of the entries of a matrix. It integrates the variance ratio  and the dimensions of the matrix by the *F *distribution function in an *ad hoc *way. Given a matrix **A**_*r*×*c *_, the SVD-p is determined as follows:

(1) Calculate the statistic  with formula

where *s_1_*, **u**_1_, and  are the leading singular value, left singular vector and right singular vector, respectively, and ||·||^2 ^represents the summation of the squares of the entries. For centralized data (row-wide, column-wide, or both), this statistic can be directly calculated by

(2) Refer  to a standard F distribution with υ_1 _= *r *+ *c *- 2 and υ_2 _= *rc *- 2*r *- *c *+ 2 as the degrees of freedom to calculate the probability of *x *> and use it as SVD-p.

While SVD-p does not hold the meaning of a p-value in a statistical test, it has the desired property of integrating the information of both cluster size and tightness. That is, the matrix of a larger cluster will have a lower SVD-p than a smaller cluster with the same tightness. The calculation is based on the assumption that, underlying a well refined gene cluster, there is a unique and exclusive pattern that can be well described by a statistical model. In this context, it is reasonable to approximate the expression matrix **A**_*r*×*c *_with a first-order bilinear equation **A **= ŝ**û **' + **Ê**, where *ŝ *is a scalar, **û **and  are *r *× *1 *and *c *× *1 *vectors respectively, and **Ê **is an r × c matrix representing the remained noise. According to Eckart-Young theorem, and Householder and Young [20], the least square estimates of the model parameters are the leading singular value, left singular vector, and right singular vector of the matrix, respectively [17,19]. There are *r + c - 2 *independent parameters in total.

### Determination of cutoffs

In Figure 2D, the two-way chart is split by two lines parallel to the x-axis and two lines parallel to the y-axis. The intercepts of these lines with the x-axis or y-axis are the cutoffs to be determined. A cutoff, like ***a***, is decided by using the following algorithm that can be easily adapted to determine other cutoffs.

(1) Assume *l*_1 _is a line parallel to the x-axis. Beginning at zero, gradually move it along the positive direction of y-axis.

(2) Calculate the SVD-p of the matrix that measures the ACG tissue and the genes mapped onto the area above the line.

(3) When the SVD-p (decreasing gradually) reaches a threshold ø (0.05 as the default), the process stops and the cutoff ***a ***is specified by the y-coordinate reading.

(4) If the threshold ø can never be reached, repeat (1) and (2) until the number of genes above the line is less than three (the assumed lower limit of the gene module size), and then decide the cutoff with the last y-coordinate reading.

As mentioned, the cutoffs determined by the algorithm are based on SVD-p. Using such cutoffs, we expect to establish a balance between the tightness (measured with ) and the size of a cluster from which gene modules are derived. This expectation holds due to the property of this statistic as mentioned above.

### Study on *M. drosophila *data

#### Data

With an in-house platform, two-color microarray data were generated from the samples of accessory gland (ACG) and malpighian tubule (MT) tissues of the W^118 ^line of *M. drosophila*, at 15, 20, 30, 45 and 60 days with two replicates at each time point. The features with more than three missing values in ten arrays of either tissue kind were excluded from further analysis, and the remaining missing values were imputed by the k-nearest neighbor algorithm [21]. Within-array and between-array normalizations were conducted using LOESS and quantile methods [22,23], respectively. The multiple probes corresponding to the same gene symbol were combined by calculating the average of their expression intensities [24]. After removing the genes that lacked change across arrays (the maximum fold-change smaller than 2), we kept about 4500 genes for further analysis. The same preprocessing procedure was performed on the two data sets independently. A more detailed description of the data sets can be found in [25] and Gene Expression Omnibus (GSE 6314).

#### Co-expression modules

**Figure 3:**
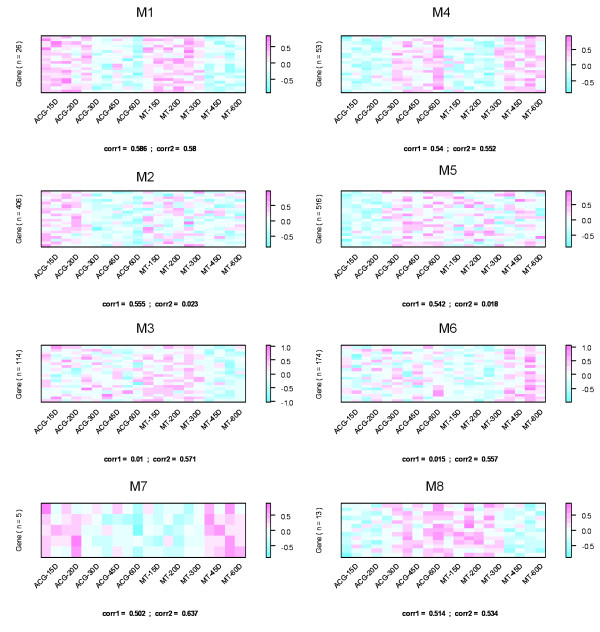
**Heatmap of eight co-expression gene modules generated from a pattern pair by svdPPCS**. **M1**: A conserved module with the down-regulation tendency appearing in both ACG data and MT data. **M2**: A divergent module with the down-regulation tendency appeared in ACG data but not MT data. **M3**: A divergent module with the down-regulation tendency appeared in MT data but not ACG data. **M4**: A conserved module with the up-regulation pattern appearing in both data sets. **M5**: A divergent module with the up-regulation tendency appearing in ACG data but not MT data. **M6: **A divergent module with the up regulation tendency appearing in MT data but not ACG data. **M7**: A divergent module with the down-regulation tendency appearing in ACG data and up-regulation tendency appearing in MT data. **M8: **A divergent module with up-regulation tendency appearing in ACG data and down-regulation tendency appearing in MT data. **corr1, corr2**: the average of the Pearson correlation coefficients among the member genes of the modules in ACG data and MT data, respectively. In plots of M1-M6, twenty randomly selected genes from the modules are mapped.

Both ACG and MT data did not exhibit a strong structure. The first two eigengenes only amounted to 31% and 37% of variance in the ACG and MT data sets, respectively (Additional file 1). The profiles of the leading eigengene in the ACG data and the second eigengene in the MT data demonstrated apparent patterns related to the aging process. A pattern pair was established because of the high correlation (r = 0.88, p < 0.01). The significance test was conducted using t-distribution. The two primary clusters (PCLs) corresponding to this pattern pair contained 2040 genes in total. These genes were mapped to a coordination system with the first left singular vector of the ACG data as the y-axis and the second left singular vector of the MT data as the x-axis. The two-way chart, split by the four lines, determined the defined SVD-p criterion (ø = 0.05). Eight co-expression gene modules, M1-M8, were identified in this way. Each of them had a unique profile across the aging process and tissue kinds as shown in Figure 3. M1 was a conserved module consisting of 23 genes. A down-regulation tendency across the ages was shown in both ACG and MT data. The average Pearson correlations among the member genes were 0.586 for ACG and 0.580 for MT. M4 was another conserved module containing 51 genes. The average Pearson correlations among the member genes were 0.540 and 0.552 for the two tissue kinds, respectively. M4 was different from M1 in that the co-expression pattern in the two data sets demonstrated an up-regulation tendency. M2, M3, M5 and M6 were four divergent modules. The number of genes within them ranged from 114 to 516. In each of these modules, the down-regulation or up-regulation patterns appeared only in one data set. The corresponding average correlations among the member genes ranged from 0.542 to 0.571. All these correlations were significant (p < 0.01). The p-values were determined on the empirical distribution of the average between-genes correlations of 500 randomly sampled null gene sets with each containing 10-500 genes. M7 and M8 were two special modules, and their sizes were relatively small. The gene expression demonstrated opposite tendencies in ACG data and MT data.

#### Gene ontology analysis

Functional enrichment analysis was conducted by using DAVID tool [26] for each of the identified gene modules. At the level l of *FDR *< 0.01, M1 - M7 had 7, 23, 31, 6, 109, 20, and 2 over-represented GO terms, respectively. The top terms (9 or less) of each module are listed in Table 1. Most significant terms for the first conserved module (M1) were under the general category of cellular component (CC). The majority of the significant terms for another conserved module (M4) were under the category of biological process (BP). The results of the divergent modules were also interesting. For example, the top GO terms of M3 included oxidoreductase activity (MF), organelle ATP synthesis coupled electron transport (BP), mitochondrial inner membrane (CC), mitochondrial respiratory chains (MF), and others. It is well known that these biological functions are related to aging and longevity of fly and other organisms [27-30]. However, the down-regulation tendency of the genes in the module was only observed in MT. This implies that the relationship between the activity of mitochondrial related genes and the aging process is tissue-specific. A similar result was suggested by our previous study using a supervised machine learning method [25].

**Table 1:** Functional enrichment analysis of the gene modules identified by svdPPCS

Category	Term	Count	pValue	FDR
M1				
CC	GO:0044444~cytoplasmic part	15	4.53E-05	6.67E-04
CC	GO:0032991~macromolecular complex	14	1.80E-04	2.64E-03
CC	GO:0005811~lipid particle	5	2.37E-03	3.44E-02
CC	GO:0043234~protein complex	11	2.83E-03	4.08E-02
CC	GO:0044446~intracellular organelle part	12	4.96E-03	7.07E-02
M2				
MF	GO:0003735~structural constituent of ribosome	42	2.73E-12	4.54E-11
CC	GO:0030529~ribonucleoprotein complex	53	1.70E-09	2.50E-08
BP	GO:0009059~macromolecule biosynthetic process	60	5.76E-09	1.03E-07
BP	GO:0006412~translation	52	1.71E-08	3.05E-07
CC	GO:0044432~endoplasmic reticulum part	15	5.71E-07	8.40E-06
CC	GO:0005783~endoplasmic reticulum	30	1.71E-06	2.51E-05
MF	GO:0005198~structural molecule activity	50	2.64E-06	4.39E-05
CC	GO:0044444~cytoplasmic part	120	4.06E-06	5.98E-05
BP	GO:0009058~biosynthetic process	78	3.50E-06	6.25E-05
M3				
MF	GO:0016491~oxidoreductase activity	26	3.75E-08	6.23E-07
BP	GO:0006091~generation of precursor metabolites and energy	21	4.74E-08	8.46E-07
BP	GO:0006118~electron transport	17	2.59E-07	4.63E-06
CC	GO:0044429~mitochondrial part	19	3.65E-06	5.37E-05
BP	GO:0042775~organelle ATP synthesis coupled electron transport	9	5.57E-06	9.96E-05
BP	GO:0006119~oxidative phosphorylation	11	3.22E-05	5.76E-04
CC	GO:0005743~mitochondrial inner membrane	11	1.19E-04	1.74E-03
CC	GO:0005740~mitochondrial envelope	12	2.15E-04	3.17E-03
CC	GO:0005746~mitochondrial respiratory chain	7	5.32E-04	7.81E-03
M4				
BP	GO:0006259~DNA metabolic process	9	7.59E-04	1.35E-02
BP	GO:0043285~biopolymer catabolic process	6	3.14E-03	5.46E-02
BP	GO:0006325~establishment and/or maintenance of chromatin architecture	6	3.32E-03	5.76E-02
BP	GO:0006323~DNA packaging	6	3.32E-03	5.76E-02
BP	GO:0006464~protein modification process	12	3.44E-03	5.97E-02
BP	GO:0043412~biopolymer modification	12	4.70E-03	8.07E-02
M5				
BP	GO:0007010~cytoskeleton organization and biogenesis	57	5.19E-11	9.28E-10
BP	GO:0030029~actin filament-based process	30	7.43E-11	1.33E-09
BP	GO:0030036~actin cytoskeleton organization and biogenesis	29	1.90E-10	3.39E-09
CC	GO:0005856~cytoskeleton	41	1.29E-08	1.90E-07
CC	GO:0044430~cytoskeletal part	34	5.62E-08	8.27E-07
CC	GO:0015629~actin cytoskeleton	20	1.49E-07	2.19E-06
BP	GO:0006996~organelle organization and biogenesis	83	1.40E-07	2.50E-06
BP	GO:0048869~cellular developmental process	85	1.43E-07	2.55E-06
BP	GO:0007015~actin filament organization	17	3.96E-07	7.08E-06
M6				
MF	GO:0032555~purine ribonucleotide binding	32	3.22E-05	5.34E-04
MF	GO:0017076~purine nucleotide binding	32	5.81E-05	9.64E-04
MF	GO:0005524~ATP binding	27	7.40E-05	1.23E-03
MF	GO:0032559~adenyl ribonucleotide binding	27	7.40E-05	1.23E-03
MF	GO:0030554~adenyl nucleotide binding	27	1.39E-04	2.30E-03
MF	GO:0016876~ligase activity, forming aminoacyl-tRNA and related component	7	2.28E-04	3.77E-03
MF	GO:0016875~ligase activity, forming carbon-oxygen bonds	7	2.28E-04	3.77E-03
MF	GO:0004812~aminoacyl-tRNA ligase activity	7	2.28E-04	3.77E-03
BP	GO:0043039~tRNA aminoacylation	7	3.02E-04	5.39E-03
M7				
BP	GO:0019752~carboxylic acid metabolic process	3	2.68E-03	4.68E-02

#### Comparison with regression analysis

As shown in Figures 2A and 2B, the biologically significant patterns hidden in both data sets were not complicated. The linear regression analysis was naturally a competitive method for identifying gene modules similar to those (M1-M8) identified by svdPPCS. For this consideration, we scanned the regression coefficients of the expression of the 4500 genes in both data sets versus the ages of the flies when the samples were taken. And then, according to the p-values (set the cutoff at 0.05) and signals (+ or -) of the coefficients of individual genes, we extracted eight gene modules (G1-G8) corresponding to the modules M1-M8. For example, G1, the counterpart of M1, consisted of the genes with p < 0.05 and negative regression coefficients in both data sets. After that, we counted the number of over-represented (FDR < 0.01) GO terms in G1-G8 as well as in M1-M8. For each module, we also calculated an information index. The index was the ratio of the number of over-represented GO terms to the number of genes. As summarized in Tables 1, 2 and Additional file 2, in general, modules identified by svdPPCS were partially overlapped with those identified using regression analysis but had comparably higher information indexes.

**Table 2:** Comparison of the results from svdPPCS and regression analysis^a^

Module	**N**_**reg**_	**N**_**svd**_	**N**_**reg-svd**_	**I**_**reg**_	**I**_**svd**_
G1/M1	25	26	12	0.08	0.28
G2/M2	255	406	196	0.09	0.06
G3/M3	146	114	73	0.24	0.27
G4/M4	27	53	17	0.04	0.11
G5/M5	352	516	306	0.19	0.21
G6/M6	173	174	102	0.03	0.15
G7/M7	8	5	2	0	0.40
G8/M8	17	13	6	0.58	0

^a^N_reg_: the number of genes in the module identified by regression analysis. N_svd_: the number of genes in the modules identified by svdPPCS. N_reg-svd_: the number of genes common in the regression module and the svdPPCS module. Ireg: the information index in the regression module. Isvd: the information index in svdPPCS module.

#### Comparison with cubic spline regression analysis plus PAM based clustering

Theoretically, the non-linear model (NLM) is more appropriate than the simple linear regression for time-series gene expression data analysis. However, its statistical power may not be guaranteed when there are limited arrays and/or time points. Storey et al. used NLM, including cubic spline regression and other methods, to analyze two time-series gene expression data sets from human and developed the software EDGE [31]. Using cubic spline regression implemented in EDGE, we identified age-related gene expression changes on ACG and MT data sets respectively. The genes with ordinary p-value less than 0.05 were stated as significant. After that, we divided these genes using PAM (Partitioning Around Medoids) clustering algorithm [32]. Based on Siliinfo average width criterion [33], the "optimal" number of clusters was two for both data sets. From the four clusters (two from ACG data and two from MT data), we extracted four divergent co-expression modules with the gene number greater than five. No conserved modules were identified. The four divergent modules contained 169, 61, 81 and 80 genes, and had 9, 47, 0 and 4 over-represented GO terms (FDR < 0.1), respectively. The GO terms in these modules are listed in Additional file 3. The well-documented age-related terms about mitochondrion and electron transport (see the section of ***Gene ontology analysis***) were not over-represented in any of these modules. It is evident that, this approach is inferior to svdPPCS and the simple linear regression analysis in terms of the number of the identified modules and the provided biological insights.

#### Application of Gap statistic as the alternative to SVD-p

Gene shaving is a special clustering method. It selects co-expression gene sets based on the first loading of the gene expression matrix or the orthogonalized matrices (after the first round). The Gap statistic on which gene shaving depends is a natural alternative to SVD-p on which svdPPCS relies. Therefore, based on the previously identified pattern pairs in the ACG and MT data sets, we calculated the Gap curves using a similar procedure documented in [13]. The results are summarized in Additional file 4. It is evident that the magnitudes of the cutoffs optimized on Gap curves are too large. Applying these cutoffs to the scatter chart in Figure 2 does not lead to informative results. In particular, among the 2040 genes mapped onto the chart, only 31 genes are above the line ***a***, 50 genes are below the line ***b***, 17 genes are on the right of the line ***c***, and 13 genes are on the left of the line ***d***. The gene modules obtained from such splitting are too small in size to provide meaningful biological insights through functional enrichment analysis. This suggests Gap statistic is not applicable to the addressed scenario.

### Simulation study

One advantage of svdPPCS over regression analysis is that the relationship between the expressions of individual genes is considered in the former, but not in the latter. In addition, svdPPCS can address a data set with more complicated patterns, such as a sine curve, which cannot be well fitted by a simple linear model. In this section, we conducted a simulation study to demonstrate the validity of svdPPCS in analyzing this kind of data.

#### Data simulation

Two data sets, "mouse" and "human" were simulated. Each of them contained 338 orthologous genes and had 18 arrays representing a set of experimental conditions (1-18 h). The noise terms of the "expression" of these genes were generated using a true microarray data set sourced from 36 samples of mouse and human stem cell [34]. All the genes were involved in cell cycle process. The arrays were divided into four groups, i.e. mouse ES (undifferentiated), mouse EB (early-differentiated), human ES, and human EB. A linear model with the species (mouse or human) and status (ES or EB) as fixed factors was implemented on each gene. By subtracting the estimated fixed effects from the expression measures, we generated the noise vector for a gene as well as the noise matrix for all the 338 genes. The noise matrix was divided into two sub-matrices and each of them contained 18 columns. Finally, two synthetic data sets were generated by adding patterns that simulated several statistical functions to the noise matrices. From the 338 genes, six classes (C1 - C6) were simulated and each class contained 30 genes. The co-expression patterns added to them are listed as follows:

C1: sine curve across the 18 time points for both mouse and human data sets.

C2: sine curve across the 18 time points for mouse data.

C3: sine curve across the 18 time points for human data.

C4: gamma(9, 2) density curve across the 18 time points for mouse data.

C5: gamma(9, 2) density curve with a time shift of 1.5 hours across the 18 time points for human data.

C6: beta(6, 6) density curve across the 18 time points for both mouse and human data sets. The other entries of the synthetic data sets had the same values as the counterparts in the noise matrices.

#### Result

**Figure 4:**
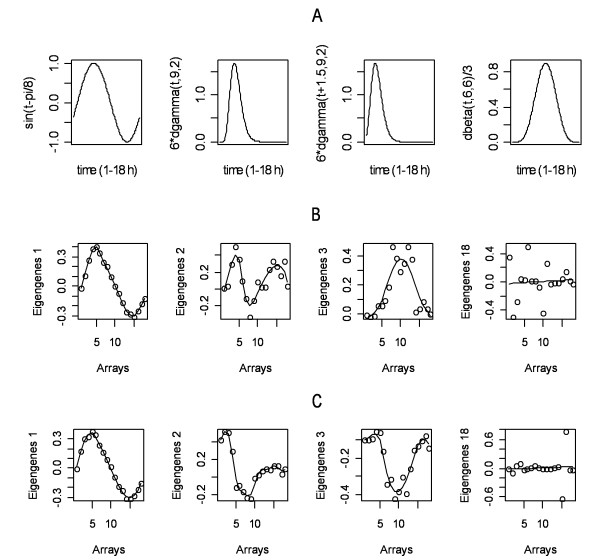
**Simulation of gene expression patterns and the recognition through the profiles of the right singular vectors (eigengenes) of the simulated human and mouse data sets**. **A**: Four statistical curves used in the simulation. **B**: The plots of the four eigengenes of the human data set versus 18 time points. **C: **The plots of the four eigengenes of the mouse data set versus 18 time points.

Figure 4A presents the four statistical curves used in the simulation. Figures 4B and 4C listed the plots of the first four eigengenes versus the time points for the human and mouse data sets, respectively. It is quite apparent that eigengene-1 and eigengene-3 recognized the patterns of the sine curve and the dbeta(t, 6,6) curve respectively in both data sets. The dgamma(t, 9, 2) curve was only simulated in the mouse data set. The eigengenene-2 of the data set corresponded approximately to the curve but demonstrated some deviation. Similarly, the dgamma(t+1.5, 9,2) curve was simulated only in the human data. The plot of the second eigengene of the data approximately recognized the structure. Based on the correlations between these eigengenes, four pattern pairs were established. They were:

Pair-1: eigengene-1 in mouse and eigengene-1 in human (r = 0.992, p < 0.01).

Pair-2: eigengene-3 in mouse and eigengene-3 in human (r = -0.873, p < 0.01).

Pair-3: eigengene-2 in mouse and eigengene-18 in human.

Pair-4: eigengene-2 in human and eigengene-18 in mouse.

Because the pattern of the eigengene-2 in human data does not match any eigengenes of the mouse data, we paired it with the eigengene-18. Eigengene-18 corresponds to an empty primary cluster (PCL) of the mouse data. The pair-4 formed due to a similar consideration.

**Figure 5:**
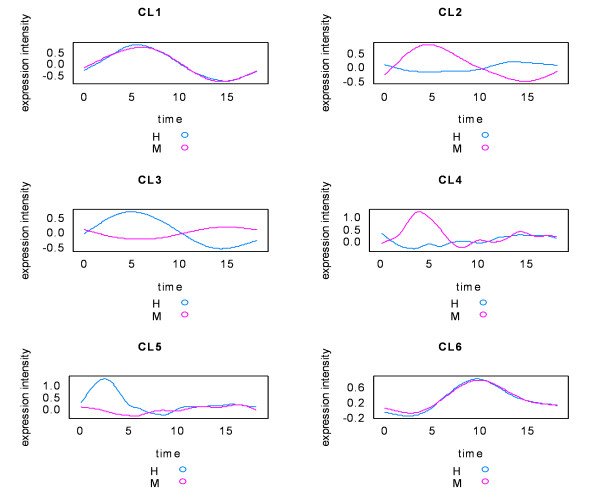
**Co-expression gene patterns of the modules from the simulated human (H) and mouse (M) time series microarray data sets**. The curves were polished using a smooth spline technique.

Using svdPPCS, we identified two major conserved co-expression gene modules (CL1 and CL6) and four divergent modules (CL2-CL5). They approximately matched the simulated gene class C1, C6, and C2-C5 as presented in Table 3 and Figure 5. It should be noted that several identified small gene modules with the size less than five were discarded.

**Table 3:** Comparison between simulated groups and identified modules

Group	Module	N1	N2	N3
C1	CL1	30	30	30
C2	CL2	30	34	24
C3	CL3	30	29	23
C4	CL4	30	30	27
C5	CL5	30	35	29
C6	CL6	30	34	30

^a^**N1**: The number of genes in the simulated group; **N2**: The number of genes in the identified module; **N3**: The numbers of genes in both the simulated group and the identified module.

#### Comparison with standard clustering algorithms

Two clustering methods, agglomerative hierarchical clustering (HC) [35] and PAM algorithm [32], were also applied to the simulated human and mouse data sets respectively. HC was trained with complete linkage and Euclidean distance as the parameters. As shown in the dendrograms (Additional file 5), this method has difficulties in correctly identifying the true structure of the simulated gene groups (three groups each carrying a unique pattern and containing 30 or 60 genes, and one null group of 218 genes) in both human and mouse data sets. On the other hand, for both data sets, the optimized number of clusters suggested by PAM based on Siliinfo average criterion is three (Additional file 6), one less than the true number of the simulated four groups. These results indicate that both clustering algorithms cannot precisely assess the determined true structures of the data sets. Therefore, compared to svdPPCS, they are not ideal candidates for this simulation study.

## Discussion

The implementation of the SVD technique for grouping genes to identify transcriptional modules is not entirely new but still active. We developed the methodology from two aspects. The first aspect is represented in the optional step to establish the primary clusters (PCLs). As a result, the gene modules identified in the subsequent steps will be largely exclusive of each other. This is important because the discovered modules can explicitly catch the main interest in some transcriptional programs such as in the *M. Drosophila *data sets analyzed in this paper. Skipping this step will lead to gene modules that can substantially overlap with each other when the modules are derived from two multiple pairs of left singular value vectors. The underlying principle of this classification is the rule of maximum confidence score adopted in supervised learning [36,37]. Each right singular vector (eigengene) represents a latent class. The projections of gene expression profiles onto the eigengene can be considered as confidence scores assigning genes to the classes. The voting mechanism is similar to that in solving a multiple classification problem [36,38].

The second aspect is the SVD-p statistic. In SVD-based algorithms, the generation or refinement of a gene cluster as well as the derived modules is based on the magnitudes of the elements of the corresponding left singular vector. A gene will be assigned to the (refined) cluster only if its value in the vector is larger (or smaller) than a threshold. The choice of the cutoff remains a major challenge in applying the related algorithms. In this study, the cutoffs are determined by a data-driven algorithm using the well-defined SVD-p statistic. It integrates the variance ratio and the dimensions of the matrix by an F-distribution function in an *ad hoc *way. Based on this formulation, the matrix of a big cluster will have a lower SVD-p than a cluster with the same tightness but a smaller size. Using SVD-p as the criterion to select the cutoffs, we established a balance between the tightness and size of a gene cluster from which gene modules are derived. The validity was verified by the analysis of the experimental data sets and the simulation study.

Biologically meaningful results were obtained by applying the proposed method to a couple of time series microarray data sets generated from the samples of accessory gland (ACG) and malpighian tubule (MT) tissues of the line W^118 ^of *M. drosophila*. Two conserved modules and six divergent modules, each of which had its unique characteristic profile across tissue kinds and aging process, were identified. The number of genes contained in these models ranged from five to a few hundred. Three to over a hundred GO terms were over-represented in individual modules with FDR < 0.1. By summarizing the results of functional enrichment analysis, we had two findings that may be important in biology. First, in one conserved module (M4) where gene expression was up-regulated across ages, several significant GO terms are involved in DNA metabolic process, DNA packaging, and the maintenance of chromosome architecture. This may suggest an anti-aging mechanism in eukaryote. Second, one divergent module (M3) showed the tissue-specific relationship between the expressions of mitochondrion-related genes and the aging process.

The comparison with other alternate methods further demonstrated the strength of svdPPCS. On the fly data sets, svdPPCS proved to be superior to linear regression analysis and cubic spline regression plus PAM based clustering in terms of the number of the identified modules and the biological insights inferred from the modules. The cutoffs determined by SVD-p were shown to be more reasonable than those decided from calculating Gap statistic with respect to the identification of co-expression gene modules of biological interest. On the simulated data sets, compared to aggregative hierarchical clustering and PAM, svdPPCS was able to perfectly identify the simulated patterns of true structures and grouped the genes with high precision.

It should be emphasized that the effectiveness of svdPPCS depends mainly on the existence of gene expression pattern(s) related to the addressed biological theme, rather than the magnitudes of the singular values corresponding to the first eigengenes. It is true that if the data do not exhibit a strong structure, the singular values are relatively small for the first eigengenes. However, svdPPCS still can perform well in such a case as actually demonstrated in the analyzed fly data. In this scenario, the first two eigengenes only accounted for 31% and 37% of variance in the ACG and MT data sets, respectively. This was an important reason why svdPPCS outperformed other alternative methods in identifying the biologically significant gene groups hidden in the data sets.

As demonstrated above, svdPPCS is a promising tool for identifying conserved and divergent co-expression modules of multiple sets of microarray experiments. However, it is worth noting that current implementation still has some limitations. First, svdPPCS requires the data sets hold comparable experimental conditions, although full equivalence is not necessary. For example, in comparing the age-related co-expression gene modules of human and mouse, we can "align" the physical ages of these two species, but such alignment may be unrealistic in comparison of mouse and fly. Second, when there are multiple patterns in the data sets or the only pattern of interest is not indicated by the leading eigengene, the generation of primary clusters (PCL) is a necessary step. In such a case, only the genes of the related primary clusters will be mapped onto the scatter chart in Figure 2D. This may cause a loss of information due to the exclusion of some genes with multiple patterns. Third, there lack "golden criteria" to decide how many primary clusters should be kept as well as if the genes with the largest projection on less important eigengenes should be reintroduced into the existing primary clusters. Further studies to rigorously address those issues are on our agenda.

## Conclusions

Using the SVD technique, we have developed a computational tool (svdPPCS) to identify conserved and divergent co-expression modules of two sets of microarray experiments. The proposed methods can be directly extended to the comparisons of multiple data sets. It is based on the assumption that under each of the co-expression patterns there is a characteristic mode [11] which can be represented by the profile of a right singular vector of the gene expression matrix. The conserved and divergent modules are identified via splitting a two-way chart defined by a pair of left singular value vectors corresponding to a pattern common or similar in the two data sets. That is, the two-way chart is divided into nine blocks with two vertical lines and two horizontal lines as four cutoffs, and the genes projected onto a single block are assigned to the same sub-cluster. We excluded the genes contained in the sub-cluster at the center of the chart from further analysis, and named the remaining non-empty sub-clusters as modules. The proposed method is generally applicable to the comparative analysis of transcriptional profiling and the integration of data sets from different platforms or of different sources, especially for the comparison of time series data sets of related organisms and different tissue kinds of the same organism under equivalent or similar experimental conditions.

## Availability and Requirements

**Project name: **svdPPCS

**Operating system: **Windows

**Programming language: **R

**License: **Free for non-commercial use. Source code available upon request.

## Authors' contributions

WZ carried out the statistical analysis and drafted the manuscript. AE, WF, DZ and KZ contributed to the method development. AE, WF, DZ and KZ provided editorial comments and participated in writing. KZ supervised and coordinated the project. All authors read and approved the final manuscript.

## Supplementary Material

Additional file 1 **Distributions of proportions of variance explained by individual eigengenes on ACG and MT data sets**. Click here for file

Additional file 2 **Functional enrichment analysis of the gene modules identified by regression analysis**. Click here for file

Additional file 3 **Functional enrichment analysis of the gene modules identified by cubic spline regression plus PAM clustering**. Click here for file

Additional file 4 **Curves of Gap statistic based on the left singular vectors of the singular value decompositions of ACG and MT data**. Click here for file

Additional file 5 **Analysis of the simulated datasets with agglomerative hierarchical clustering algorithm**. Click here for file

Additional file 6 **The optimal number of clusters suggested by PAM algorithm and Siliinfo average width criterion on the simulated data sets**. Click here for file
