# Supplementary material for: svdPPCS: an effective singular value decomposition-based method for conserved and divergent co-expression gene module identification
Source: BMC Bioinformatics. 2010 Jun 22;11:338. doi: 10.1186/1471-2105-11-338 (PMC2905369; doi:10.1186/1471-2105-11-338)
Supplement: Additional file 2 — Functional enrichment analysis of the gene modules identified by regression analysis. [file 1471-2105-11-338-S2.PDF]

Additional file 2: Functional enrichment analysis of the gene modules identified by regression analysis

Category	Term	Count	pValue	FDR
G1				
CC	GO:0044444~cytoplasmic part	11	3.49E-04	5.13E-03
CC	GO:0031090~organelle membrane	6	1.62E-03	2.36E-02
G2				
CC	GO:0005783~endoplasmic reticulum	26	3.72E-09	5.48E-08
CC	GO:0044432~endoplasmic reticulum part	12	7.31E-07	1.08E-05
CC	GO:0042175~nuclear envelope-endoplasmic reticulum network	10	4.68E-06	6.90E-05
CC	GO:0005789~endoplasmic reticulum membrane	10	4.68E-06	6.90E-05
BP	GO:0045047~protein targeting to ER	7	1.44E-05	2.57E-04
BP	GO:0006614~SRP-dependent cotranslational protein target…	7	1.44E-05	2.57E-04
BP	GO:0006613~cotranslational protein targeting to membrane	7	2.74E-05	4.89E-04
BP	GO:0006612~protein targeting to membrane	7	4.83E-05	8.63E-04
CC	GO:0005737~cytoplasm	84	6.57E-05	9.67E-04
G3				
MF	GO:0016491~oxidoreductase activity	31	2.11E-08	3.50E-07
CC	GO:0044429~mitochondrial part	22	3.27E-06	4.82E-05
BP	GO:0006091~generation of precursor metabolites and energy	22	3.10E-06	5.54E-05
CC	GO:0005739~mitochondrion	25	7.82E-06	1.15E-04
BP	GO:0006118~electron transport	16	8.94E-05	1.60E-03
BP	GO:0006119~oxidative phosphorylation	12	1.08E-04	1.92E-03
BP	GO:0042775~organelle ATP synthesis coupled electron …	8	4.32E-04	7.69E-03
BP	GO:0042773~ATP synthesis coupled electron transport	8	5.90E-04	1.05E-02
CC	GO:0005761~mitochondrial ribosome	8	7.71E-04	1.13E-02
G4				
BP	GO:0051246~regulation of protein metabolic process	5	4.77E-04	8.49E-03
G5				
CC	GO:0005856~cytoskeleton	31	2.31E-07	3.41E-06
BP	GO:0007010~cytoskeleton organization and biogenesis	38	2.11E-07	3.78E-06
MF	GO:0005515~protein binding	185	1.71E-06	2.84E-05
MF	GO:0005200~structural constituent of cytoskeleton	21	1.99E-06	3.31E-05
MF	GO:0008092~cytoskeletal protein binding	25	5.12E-06	8.50E-05
BP	GO:0030036~actin cytoskeleton organization and biogenesis	18	6.49E-06	1.16E-04
BP	GO:0000902~cell morphogenesis	38	7.34E-06	1.31E-04
BP	GO:0032989~cellular structure morphogenesis	38	7.34E-06	1.31E-04
BP	GO:0002009~morphogenesis of an epithelium	20	7.80E-06	1.39E-04
G6				
MF	GO0016876~ligase activity, forming aminoacyl-tRNA and…	6	1.57E-03	2.57E-02
MF	GO:0016875~ligase activity, forming carbon-oxygen bonds	6	1.57E-03	2.57E-02
MF	GO:0004812~aminoacyl-tRNA ligase activity	6	1.57E-03	2.57E-02
CC	GO:0005622~intracellular	83	2.12E-03	3.08E-02
CC	GO:0044424~intracellular part	78	4.19E-03	5.99E-02
BP	GO:0009059~macromolecule biosynthetic process	21	4.72E-03	8.11E-02
G8				
BP	GO:0000226~microtubule cytoskeleton organization and …	4	7.60E-04	1.35E-02
BP	GO:0048513~organ development	7	1.10E-03	1.94E-02
BP	GO:0048856~anatomical structure development	8	1.71E-03	3.01E-02
BP	GO:0032502~developmental process	9	2.22E-03	3.89E-02
BP	GO:0048731~system development	7	3.92E-03	6.78E-02
BP	GO:0007154~cell communication	7	4.10E-03	7.08E-02
BP	GO:0007242~intracellular signaling cascade	5	4.62E-03	7.93E-02
BP	GO:0007275~multicellular organismal development	8	4.86E-03	8.33E-02
BP	GO:0007010~cytoskeleton organization and biogenesis	5	5.45E-03	9.29E-02
