# Supplementary material for: svdPPCS: an effective singular value decomposition-based method for conserved and divergent co-expression gene module identification
Source: BMC Bioinformatics. 2010 Jun 22;11:338. doi: 10.1186/1471-2105-11-338 (PMC2905369; doi:10.1186/1471-2105-11-338)
Supplement: Additional file 3 — Functional enrichment analysis of the gene modules identified by cubic spline regression plus PAM clustering. [file 1471-2105-11-338-S3.PDF]

Additional file 3: Functional enrichment analysis of the gene modules identified by cubic spline regression plus PAM clustering

Category	Term	Count	PValue	FDR %
ACG-module1 (divergent)				
CC	GO:0044430~cytoskeletal part	13	2.78E-04	0.34
BP	GO:0007010~cytoskeleton organization	15	4.52E-04	0.70
CC	GO:0005856~cytoskeleton	14	4.95E-04	0.61
MF	GO:0005515~protein binding	77	0.0020889	2.72
BP	GO:0006996~organelle organization	21	0.0029777	4.52
CC	GO:0043228~non-membrane-bounded organelle	23	0.0032813	3.98
CC	GO:0043232~intracellular non-membrane-bounded organelle	23	0.0032813	3.98
CC	GO:0015629~actin cytoskeleton	6	0.0051208	6.15
ACG-module2 (divergent)				
BP	GO:0060429~epithelium development	8	1.60E-05	0.02
BP	GO:0007010~cytoskeleton organization	10	2.61E-05	0.04
BP	GO:0065008~regulation of biological quality	11	2.74E-05	0.04
BP	GO:0035150~regulation of tube size	4	7.24E-05	0.11
BP	GO:0002009~morphogenesis of an epithelium	7	1.30E-04	0.19
BP	GO:0048729~tissue morphogenesis	7	2.17E-04	0.32
BP	GO:0035152~regulation of tube architecture, open tracheal system	4	3.32E-04	0.48
BP	GO:0016043~cellular component organization	16	5.09E-04	0.74
BP	GO:0008154~actin polymerization or depolymerization	3	6.83E-04	0.99
CC	GO:0043296~apical junction complex	4	7.31E-04	0.83
Mt-module1 (divergent)				
BP	GO:0008610~lipid biosynthetic process	6	3.00E-04	0.43
BP	GO:0044255~cellular lipid metabolic process	6	0.0021001	2.98
BP	GO:0016053~organic acid biosynthetic process	4	0.0039675	5.55
BP	GO:0046394~carboxylic acid biosynthetic process	4	0.0039675	5.55
Mt-module2 (divergent)	No term with FDR < 10%			
