# Supplementary material for: svdPPCS: an effective singular value decomposition-based method for conserved and divergent co-expression gene module identification
Source: BMC Bioinformatics. 2010 Jun 22;11:338. doi: 10.1186/1471-2105-11-338 (PMC2905369; doi:10.1186/1471-2105-11-338)
Supplement: Additional file 4 — Curves of Gap statistic based on the left singular vectors of the singular value decompositions of ACG and MT data. [file 1471-2105-11-338-S4.PDF]

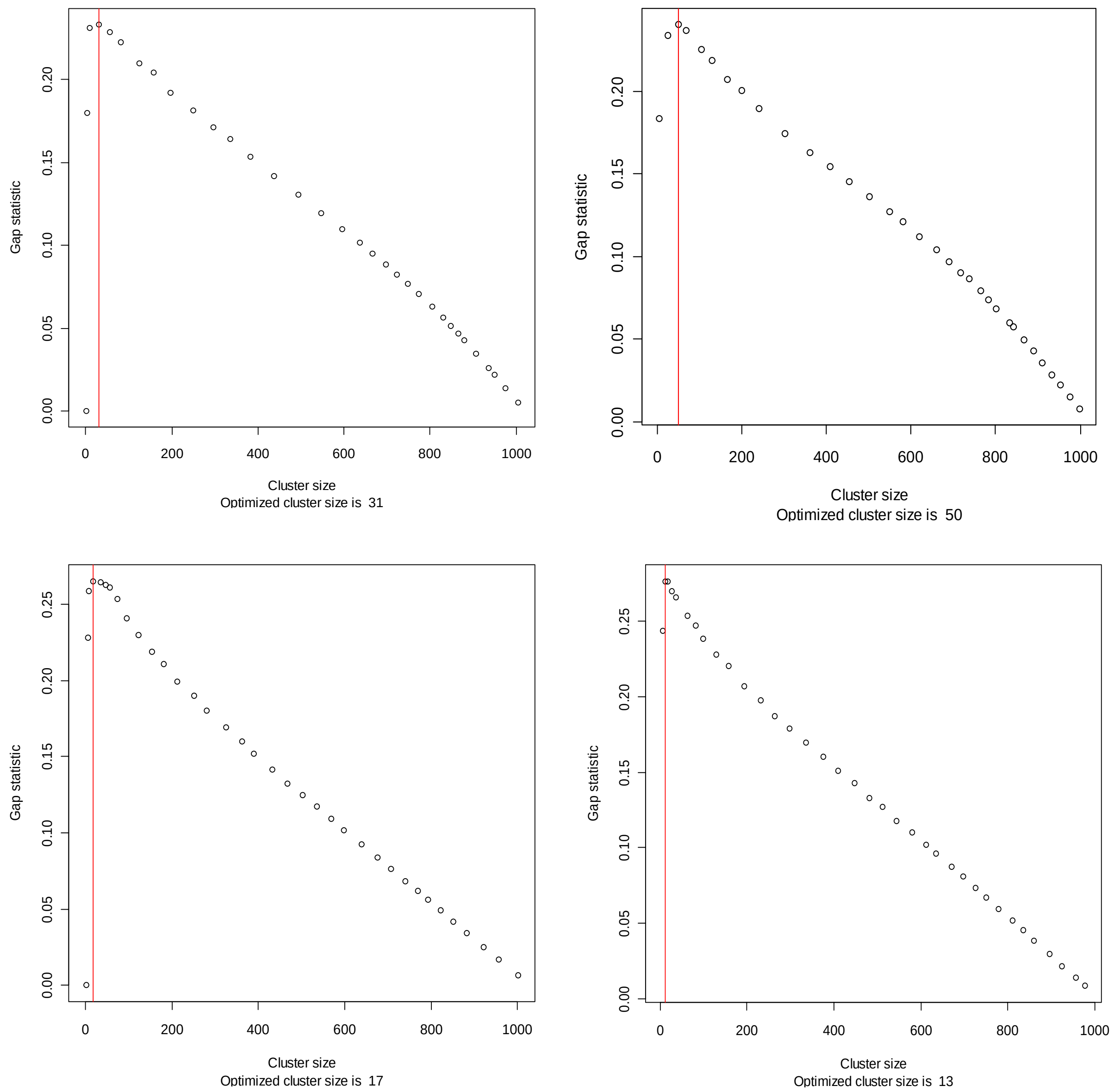

Additional file 4: Curves of Gap statistic for gene shaving algorithm based on the left singular vectors of the singular value decompositions of ACG and MT data. **Top left**: positive orientation of the first left singular vector of ACG data set. **Top right**: negative orientation of the first left singular vector of ACG data set. **Bottom left**: positive orientation of the second left singular vector of MT data set. **Bottom right**: negative orientation of the second left singular vector of MT data set.
