# Supplementary material for: svdPPCS: an effective singular value decomposition-based method for conserved and divergent co-expression gene module identification
Source: BMC Bioinformatics. 2010 Jun 22;11:338. doi: 10.1186/1471-2105-11-338 (PMC2905369; doi:10.1186/1471-2105-11-338)
Supplement: Additional file 6 — The optimal number of clusters suggested by PAM algorithm and Siliinfo average width criterion on the simulated data sets. [file 1471-2105-11-338-S6.DOC]

**Additional file 6:** The optimal number of clusters suggested by PAM algorithm and Siliinfo average width criterion
